# Ameliorative effects of aerobic training in girls with precocious puberty: role of leptin and ghrelin

**DOI:** 10.1038/s41598-023-42828-1

**Published:** 2023-09-21

**Authors:** Ramyar Najafi, Ali Heidarianpour, Elnaz Shokri, Behnaz Shokri

**Affiliations:** Faculty of Sport Sciences, Bu-Ali Sina University, Hamedan, Iran

**Keywords:** Physiology, Endocrinology

## Abstract

This study was an attempt to examine the changes in serum levels of ghrelin and leptin after 12-weeks of aerobic training and gonadotropin releasing hormone agonist (GnRH) treatment in girls with central precocious puberty. Thirty girls (6–8 years old) with precocious puberty who had received Triptorelin were randomly divided in two groups (medication and medication + training). Fifteen age-matched healthy girls (without precocious puberty) were also included as the control group. The medication + training group submitted an aerobic training program for 3 days/week with 20–75 min per day and 45–75% of maximum heart rate for 12-weeks. Serum levels of leptin, ghrelin, cholesterol, triglycerides and body mass index (BMI) were determined at baseline and 48 h after the last training session. The results indicated that leptin significantly decreased (*p* = 0.001) and ghrelin significantly increased (*p* = 0.001) in the medication + training group but no significant difference was observed in the ghrelin (*p* = 1) and leptin (*p* = 0.78) in the medication group. Leptin to ghrelin ratio indicated a decrease in medicine + training group (*p* = 0.028). Ghrelin were negatively correlated with leptin and BMI. The data indicated that aerobic training increased ghrelin and reduced leptin and leptin to ghrelin ratio but GnRH agonist treatment had no effect on plasma leptin and ghrelin levels.

## Introduction

Puberty is a vital process in the development of all individuals. The series of hormonal changes during puberty result in the physical development of sexually mature adults. In addition to sexual maturity children also go through other physical and emotional changes such as hair growth, voice changes, and acne^1^. Puberty usually occurs in girls between the ages of 10 and 13. If it begins before the age of 8 in girls, it is defined as precocious puberty^2^. Research shows that the prevalence of precocious puberty is increasing. In 2010, prevalence of central precocious puberty (CPP) was 55.9 per 100,000 children in girls and 1.7 per 100,000 children in boys in Asia^3^. In 2014, prevalence of CPP was 193.2 per 100,000 person^4^. The choice treatment for CPP is Gonadotropin releasing hormone (GnRH) agonist^5^. Initially, GnRH stimulates the synthesis and secretion of Luteinising hormone (LH) and Follicle-stimulating hormone (FSH), but when it is administered chronically, GnRH suppresses the production of these hormones, which, in turn, suppresses the production of sex steroid hormones by the gonads^6^. Although the benefits of GnRH therapy in terms of improving adult height in girls older than age 7 at the start of treatment has been questioned^7^.

Obesity has been shown to impact the timing of puberty and may be among the causes for the earlier trends of pubertal age reported in various countries^8^. Brix et al. found that higher childhood body mass index (BMI) was associated with earlier pubertal timing in boys and girls in both a cohort analysis and a sibling-matched analysis^9^. The results of a meta-analysis conducted by Li et al. In 2017 showed that the number of girls with early puberty was significantly higher in the obese group than the normal weight group^10^. Also, in girls with CPP, weight gain has been reported after treatment with GnRHa^11^. Excessive positive energy balance is a major factor leading to obesity and therefore premature puberty. The ability to alter the appetite-regulating hormones may help decrease excessive energy intake^12^. Leptin is one of the most relevant appetite-regulating hormones. In addition, it may well be one of the hormonal factors that signal to the brain the body’s readiness for sexual maturity and reproduction^13^. Some studies show that the serum leptin levels in girls with CPP are higher than those of normal pubertal controls^14^. Another appetite-regulating hormone is ghrelin. Ghrelin, a peptide hormone identified in the stomach, is directly involved in the regulation of energy balance and obesity^15^. There is evidence showing that ghrelin may affect the reproductive function in animals and humans by decreasing pituitary LH secretion^16^. During puberty, a progressive reduction in ghrelin levels has been reported^17^. Ghrelin was inversely associated with leptin in girls^18^. Therefore, it can be theorized that evaluating the leptin to ghrelin ratio can be a more efficient assessment of the results of appetite-regulating hormone status.

Studies showed that leptin levels did not differ from the values after 6 months of GnRH agonist administration^11^. But physical exercise has been used as a non-pharmacological tool in management of body weight and the effect of physical activity on weight control is an important issue for clinical studies in the field of endocrinology^15^. Short-term exercise (60 min) in obese females did not alter leptin concentrations but exercise training protocols (12-weeks) that result in reduced fat mass are generally accompanied by lower leptin concentrations^19^. Investigations have demonstrated that exercise training increases total ghrelin levels in adolescents^20^. So far, no study has evaluated the effect of exercise on leptin, ghrelin and leptin to ghrelin ratio in precocious puberty girls. Therefore, the present study tries to fill this gap.

## Materials and methods

The statistical population of this term non-randomized prospective study included all children with precocious puberty who referred to Endocrinologist, and their precocious puberty was confirmed based on the following methods:

Criteria for detecting precocious puberty are divided into two categories: clinical features and biochemical characteristics. Clinical features in girls usually present with both breast development and pubic hair which is evaluated according to the five Tanner categorizations^21^. Uterine and ovarian enlargements are consistent with precocious puberty because uterine growth reflects estrogen stimulation. Uterine lengths > 3.5–4 cm and ovarian volumes > 2 mL is consistent with puberty^22^. Luteinizing hormone (LH) is the best biochemical parameter used to diagnose CPP. An increase in levels of LH greater than 0.2 IU/L can be considered a pubertal value^23^. Provocative test with GnRH agonist while precise cutoffs are difficult to establish, a peak stimulated LH of > ∼8 mIU/mL after GnRH and > ∼5 IU/L after GnRHa are considered indicative of CPP^5^. All these tests and examinations were performed by an endocrinologist, radiologist and laboratory officials.

**Figure 1:**
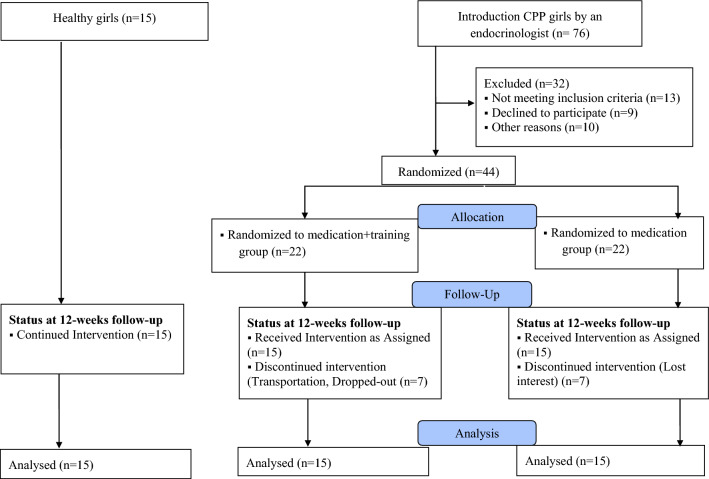
Flow diagram of the study.

Among all the girls who referred to an endocrinologist and their precocious puberty was confirmed (according to the mentioned criteria), 76 girls aged 6–8 years old were introduced to us. Of these, 30 were finally analyzed according to Fig. 1.

Inclusion criteria for the study included: 1. the onset of puberty in girls is before 8 years old; 2. All girls whit precocious puberty should use Triptorelin drug; 3. Taking medication 80 mg/kg (max: 3.75 mg) every 28 day.

The exclusion criteria in the study included: 1. having another illness; 2. taking another medicine; 3.Being active in another sport 4. Parents/patients unwilling to participate in the study.

Parents or legal guardians of all participants completed informed consent and questionnaire regarding the health status, injuries and level of activity (training) of the subjects. The subjects were randomly divided into two groups: Group 1 included the patients treated only with the GnRH agonist (80 mg/kg of Triptorelin every 28 day) through intramuscular injection, and Group 2 was treated with GnRH agonist (80 mg/kg of Triptorelin every 28 day) through intramuscular injection + aerobic training. Since, due to ethical issues, we could not leave any child with early maturity untreated, the control group of this study selected from homogeneous children without precocious puberty (healthy) (n = 15).

The research was approved by the Medical Ethics Committee of Hamedan University of Medical Sciences on 14th of Nov, 2015 with proprietary ID IR.UMSHA.REC.1394.366. We confirm that all methods were performed in accordance with the relevant guidelines and regulations.

### Measuring basic indicators

The height and weight of the subjects were measured by children stadiometer of the German SSA 216 model and beaver digital balance GS20 model, respectively. Heart rate was measured by Polar heart rate counter. BMI was analyzed based on CDC (Centers for Disease Control reference). The BMI formula for children and adults is the same (kg/m^2^), but the interpretations are different from the adult body composition index, in this way, children who are in before the 5th percentile are considered thin, children who are between the 5 and 85th percentile are balanced, children who are between the 85 and 95th percentile are overweight and children who are higher than 95th percentile are obesity^24^. Waist circumference (WC) was measured using an inextensible measuring tape at a point midway between the costal margin and the iliac crest (anterosuperior iliac spine) with the subject standing and breathing out. Hip circumference was measured at the gluteal prominence, and the waist/hip ratio (WHR) was calculated.

### Measuring blood samples

At the baseline, the subjects were pre-tested. To measure biochemical variables 24 h before the training program, blood sampling was carried out by a laboratory specialist in the morning and after 12 h of fasting; 6 cc of blood samples was obtained from the participants. Diagnostics company kit of Canada with 0.5 ng/ml degree of sensitivity and the Elisa method were used to measure leptin serum levels. To measure ghrelin serum levels, we used Estbiopharm company kit of Canada with 0.5 ng/ml degree of sensitivity and the Elisa method. Serum levels of total cholesterol (TC) and triglycerides (TG) were measured by enzymatic procedures. The samples were measured again after 12 weeks of exercise training. It should be noted that, to equalize the conditions and to eliminate the effect of the last training session, the measurements were performed 48 h after the end of the training. BMI was measured before and after program completion.

### Exercise training protocol

All the steps of aerobic training were performed in a gym under the supervision of two experienced physical training instructors. The intensity and duration of the training program as determined by the opinions of sports experts in childhood. The protocol was conducted for 12 weeks, three sessions per week. The training program consisted of three sections: warm-up, the main stage and cool-down. The warm-up included stretching, low-intensity running for 15 min. In the main stage, according to Table 1, subjects performed the increasing activity in this way: every two weeks, 5% of the hearth rate was added to the intensity of activity (220-age) and 5 min was added to activity time. Training intensity started from 45 to 50% of maximum heart rate in the first week, and at the end of the 12th week, the intensity of the training reached 70–75% of the maximum heart rate. Regarding the age and physiological abilities and other conditions of the subjects, an attempt was made to use such activities as rhythmic movements, childish games, and poetry as they create a sense of competition. In the end, 10 min of recovery including walking and slow stretching exercises within 30% of heart rate. During physical activity, the heart rate of the subjects was repeatedly measured by Polar heart rate counter and checked to match Table 1.

**Table 1:** Training protocol for 12 weeks.

Weeks	Intensity percent (MHR)	Time (minute)
1	40–50	25
2	40–50	25
3	50–55	30
4	50–55	30
5	55–60	35
6	55–60	35
7	60–65	40
8	60–65	40
9	65–70	45
10	65–70	45
11	70–75	50
12	70–75	50

### Data analysis method

The Shapiro–Wilk test was used to determine the normal distribution of the data.Analysis of covariance (ANCOVA) was used to compare the difference between groups. The Pearson correlation coefficient used to analyze the relationship between ghrelin and leptin, leptin and BMI, ghrelin and BMI. The collected data were analyzed using the SPSS 20 software. The data are presented as mean ± standard deviation and, the statistical significance was set at *P* < 0.05.

## Results

Table 2 showed specifications of subjects in the three groups (medicine, medicine + training, and control) and two stages (baseline, post-test). Data showed that the medicine, medicine + training groups were similar in terms of physiological indices and no significant difference was found between them in baseline stage. The results further showed that girls with precocious puberty have higher height, weight, BMI and cholesterol than healthy girls. In addition, weight, BMI and cholesterol levels decreased significantly after 12 weeks in the medicine + training group (*P* = 0.02, *P* = 0.01, *P* = 0.02 respectively). But there was no significant change in the control and medicine groups.

**Table 2:** Specifications of subjects in all three groups in two stages: baseline and post.

Variable	Medication group N = 15		Medication + training group N = 15		Control group N = 15	
	Baseline	Post	Baseline	Post	Baseline	Post
Height (cm)	128 ± 1.61^b^	128.7 ± 1.63^b^	127.8 ± 2.1^b^	127.9 ± 2.5^b^	123.4 ± 1.6	124.5 ± 1.3
Weight (kg)	29.5 ± 1.03^b^	30 ± 1.25^b^	29.9 ± 1.52^b^	27.8 ± 1.06^a^^,b,c^	26.3 ± 1.09	26.4 ± 1.12
BMI (kg/m^2^)	17.8 ± 0.68^b^	17.9 ± 0.72^b^	17.9 ± 0.6^b^	17.1 ± 0.44^a^^,b,c^	16.5 ± 0.49	16.6 ± 0.29
Height SDS	0.86 ± 0.07	0.89 ± 0.17	0.74 ± 0.03	0.75 ± 0.06	0.23 ± 0.14	0.31 ± 0.08
BMI SDS	0.91 ± 0.05	0.91 ± 0.22	0.94 ± 0.16	0.45 ± 0.2	0.11 ± 0.07	0.13 ± 0.09
Cholesterol (mg/dl)	195.5 ± 8.5^b^	197.2 ± 5.1^b^	196.5 ± 6.1^b^	190.8 ± 6.6^a^^,b,c^	177.6 ± 10.5	177.7 ± 11
Triglyceride (mg/dl)	123.1 ± 6.3	124.7 ± 5.4	123.7 ± 4.8	118.6 ± 5/2^a^	118.4 ± 6.2	118.5 ± 5.5
WHR	0.89 ± 0.27	0.9 ± 0.33	0.9 ± 0.16	0.87 ± 0.06	0.85 ± 0.06	0.86 ± 0.11
Age (year)	7.21 ± 1.25		7.27 ± 0.57		7.22 ± 0.48	

Data are reported as Mean ± SD.

^a^Significantly different from baseline.

^b^Significantly different with control group.

^c^Significantly different with medicine group.

*BMI* body mass index, *SDS* standard deviation score, *WHR* waist/hip ratio.

Table 3 showed the result of ANCOVA for leptin and ghrelin in the three groups (medicine, medicine + training, and control) in the two stages (baseline, post-test). A group difference in leptin change score was found to be statistically significant (F = 14.45; *P* = 0.001). Post hoc analyses revealed that leptin levels were significantly lower in the medicine + training compared to the medicine (*P* = 0.01). Leptin levels were significantly higher in the medicine group compared to control group (*P* = 0.01). No significant difference, however, in leptin concentration was found in the medicine + training group compared to control group (*P* = 0.87) (see Table 3).

**Table 3:** Result of Analysis of Covariates and Post-Hoc Pairwise comparisons for leptin and gherlin in all three groups in two stages: baseline and post.

Variable	Medication group (1)		Medication + training group (2)		Control group (3)		Analysis of covariates							Post-Hoc pairwise comparisons		
	Baseline	Post	Baseline	Post	Baseline	Post	Source	SS	df	MS	F	*P*	Power	Pair comparison of groups	Mean difference	*P*
Leptin (ng/ml)	7.93 ± 1.44	7.54 ± 1.12	7.8 ± 1.58	6.33 ± 1.61	6.2 ± 1.35	6.31 ± 1.38	Group	12.88	3	4.29	14.45	0.001	1	1–2	− 0.96	**0.01**
														1–3	− 0.95	**0.01**
							Erorr	16.33	55	0.29						
														2–3	0.01	0.87
Gherlin (ng/ml)	8.34 ± 1.21	8.4 ± 1.05	8.21 ± 1.4	9.11 ± 1.35	9.88 ± 1.3	9.9 ± 1.25	Group	16.39	3	5.46	17.33	0.001	1	1–2	1.11	**0.01**
														1–3	1.39	**0.02**
							Erorr	17.34	55	0.31						
														2–3	0.28	**0.06**

Significant values are in [bold].

There was a significant group difference in ghrelin concentration after 12 weeks of aerobic training (F = 17.33; *P* = 0.0001). Post hoc analyses revealed that ghrelin levels were significantly higher in the medicine + training group compared to the medicine group (*P* = 0.01). Ghrelin levels were significantly lower in the medicine group compared to control group (*P* = 0.02). Also, significant difference was found between the medicine + training and control group (*P* = 0.06) (see Table 3).

**Figure 2:**
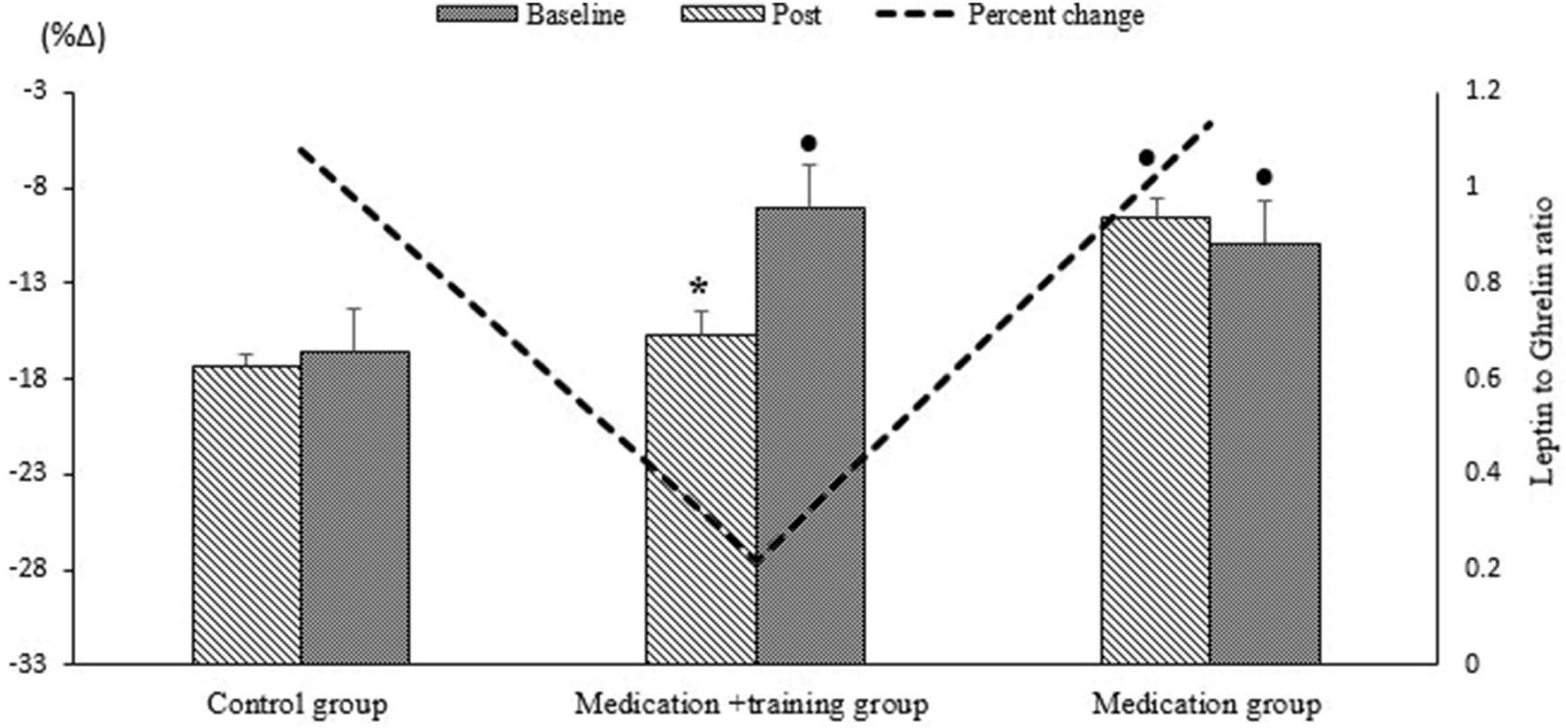
Comparison of Leptin to Ghrelin ratio and percentage of changes in 3 group (medicine, medicine + training, control) and the two stages (baseline, post-test). * Significantly different from baseline, • significantly different with control group.

Figure 2 shows the leptin to ghrelin ratio and its percentage of changes in three groups (medicine, medicine + training, and control) and two stages (baseline, post-test). The results indicate a decrease in leptin to ghrelin ratio in the medicine + training group after 12 weeks aerobic exercise (*p* = 0.028).

In addition, the results revealed that change in serum ghrelin levels were negatively correlated with change in leptin levels (r =  − 0.36, *p* = 0.003). There was a positively correlation between change in serum leptin and chane in BMI (r = 0.35, *p* = 0.01). Likewise, change in serum ghrelin levels were negatively correlated with change in BMI (r =  − 0.41, *p* = 0.02). While changes in leptin to ghrelin ratio were not significantly correlated with any of the parameters (see Table 4).

**Table 4:** Correlation between parameter changes in the baseline stage and after 12 weeks aerobic training.

Parameters	Δ leptin/ghrelin		Δ Leptin (µg/ml)		Δ Ghrelin (ng/ml)	
	r	*P*	r	*P*	r	*P*
Δ Weight (kg)	0.11	0.53	0.33	0.001*	− 0.32	0.01*
Δ BMI (kg/m^2^)	0.24	0.66	0.35	0.01*	− 0.41	0.002*
Δ Cholesterol (mg/dl)	0.14	0.1	0.25	0.03*	− 0.3	0.008*
Δ Triglyceride (mg/dl)	0.09	0.208	0.21	0.01*	− 0.15	0.46
Δ leptin					− 0.36	0.003*

*Δ* value at Post-value at baseline, *BMI* body mass index, * significant correlation.

Significant values are in [bold].

## Discussion

The major findings of this study were: (1) Leptin levels decreased significantly after aerobic training + GnRH agonist treatment. (2) Ghrelin levels increased significantly after aerobic training + GnRH agonist treatment. (3) The GnRH agonist alone did not have a significant effect on leptin and ghrelin during 12 weeks. (4) Leptin to ghrelin ratio decreased after aerobic training + GnRH agonist treatment.

To the authors’ knowledge, the present study is the first study to evaluate the effect of aerobic training on leptin, ghrelin and leptin/ghrelin ratio in precocious puberty girls. In connection with the first finding; A review article related to leptin showed that leptin decreased significantly after long-term endurance training (≥ 12 weeks), and the decreases in leptin concentrations were associated with weight loss. Exercise training protocols that lead to reduced fat mass, weight and BMI are generally accompanied by lower leptin concentrations^19^. Our results showed that body mass index, cholesterol and triglyceride levels decreased in response to aerobic training in 12 weeks in medicine + training, which can be a possible mechanism for a decrease in circulating levels of leptin in this study. Furthermore, several clinical studies have shown a correlation between increased plasma levels of both C-reactive protein and leptin^25^. Research that has already been published by us showed that C-reactive protein decreased in girls with precocious puberty after 12 weeks of aerobic training^26^. So another possible mechanism for decrease leptin levels is the reduction in inflammatory marker such as C-reactive protein.

The second finding of the study was that 12 weeks of training + GnRH agonists led to a significant increase in ghrelin levels in girls with CPP. Exercise training increases total ghrelin levels in adolescents and ghrelin is sensitive to reductions in body fat or increases in energy expenditure in adolescents^20^. Many researchers suggest that lowering weight as a result of endurance training are the main mechanisms for increasing circulating levels of ghrelin^27, 28^. In the present study, reduced weight were the main causes of increased ghrelin levels. About the possible mechanisms of the effect of weight loss on ghrelin, it can be mentioned that losing weight modifies ghrelin rhythms^29^. Also, Briggs et al.^30^ suggested hypothalamic inflammation as a potential mechanism of ghrelin resistance. Therefore, since weight loss reduces inflammatory factors^26^, the weight reduction observed in this study could have reduced local inflammation and partially restored ghrelin resistance. Although, in the present work, we did not measure LH and FSH changes, but previous studies showed that ghrelin reduces both LH and FSH. So, it can be concluded that the increase of ghrelin in the Medication + training group is probably associated with the decrease of LH and FSH. In addition to our previously published research showed that both types of aerobic and combined training reduce the signs of puberty (LH, FSH, bone age, uterine length, and ovarian volume)^31, 32^.

The third finding of the present study was that leptin and ghrelin levels did not change during GnRH treatment. In line with our results, Yoo et al. suggest that GnRH treatment in girls with CPP does not affect serum levels of leptin^11^. In adult women, suppression of the H-P-G axis by GnRHa does not affect serum leptin levels and serum leptin levels are merely associated with weight and BMI^11^. Since there was no significant change in weight, BMI, cholesterol and triglyceride levels in the medicine group, it can be the possible cause for no change in leptin levels. Studies on humans and experimental models suggest that therapeutic application of GnRH or administration of agonists thereof has the secondary effect of increasing body weight^33^. This subsequent effect has been reported in humans mainly in treatments for precocious puberty using GnRH agonists^34^, as well as cancer^35^ and fertility regulation^36^. The present study also showed that girls with precocious puberty who are treated with GnRH agonists have higher height, weight and BMI than healthy girls of the same age. Contrary to our results, Maffeis et al. showed that ghrelin circulating levels decreased significantly during treatment with GnRHa for 18 months^37^. The reasons that justify these results are not known. They stated that it is unlikely that small changes in BMI z-scores, which are not statistically significant, could explain ghrelin changes during GnRH analog treatment and estrogen may play a potential role in the regulation of ghrelin secretion^38^.

The remarkable point is that GnRHa treatment did not affect ghrelin and leptin levels, but training (or weight loss due to training) improves the metabolic profile (Leptin and ghrelin levels). Therefore, according to this finding, it is recommended that girls with CPP participate in aerobic training programs in addition to consuming GnRH.

The fourth finding of this study was that leptin to ghrelin ratio decrease significantly in the medicine + training group (*p* = 0.028). This reduction was about 27%.

## Conclusion

Based on the findings, it can be concluded that moderate intensity training that was performed for a long time and regularly is likely to reduce BMI and leptin and increase ghrelin while treatment with GnRH did not change BMI and the levels of leptin and ghrelin in girls with precocious puberty. In fact, exercise may have beneficial effects on girls with precocious puberty through weight loss. However well-controlled research is required to determine the influence of leptin and ghrelin changes regarding the suppression of the central pubertal axis.

## Data Availability

The datasets generated during and/or analyzed during the current study are available from the corresponding author on reasonable request.
